# Inoculation of black turtle beans (*Phaseolus vulgaris*) with mycorrhizal fungi increases the nutritional quality of seeds

**DOI:** 10.1002/pei3.10128

**Published:** 2023-11-15

**Authors:** Joseph E. Carrara, Lavanya Reddivari, Wade P. Heller

**Affiliations:** ^1^ USDA Agricultural Research Service Eastern Regional Research Center Wyndmoor Pennsylvania USA; ^2^ Department of Food Science Purdue University West Lafayette Indiana USA

**Keywords:** agriculture, anthocyanins, arbuscular mycorrhizae, black beans, nutrient uptake

## Abstract

The use of arbuscular mycorrhizal fungi (AMF) as biofertilizers has proven successful in boosting the yield and nutritional quality of a variety of crops. AMF associate with plant roots and exchange soil nutrients for photosynthetically derived C in the form of sugars and lipids. Past research has shown that not all AMF species are equal in their benefit to nutrient uptake and crop health, and that the most beneficial AMF species appear to vary by host species. Although an important human food staple, especially in developing regions where nutrient deficiency is a prevalent threat to public health, little work has been done to test the effectiveness of AMF in enhancing the nutritional quality of common bean (*Phaseolus vulgaris L.*). Therefore, our objective was to determine the most beneficial AMF species for inoculation of this important crop. We inoculated black beans (*Phaseolus vulgaris* black turtle beans) with eight individual AMF species and one mixed species inoculum in an outdoor pot trial over 3 months and assessed the extent to which they altered yield, mineral nutrient and anthocyanin concentration of seeds and leaf tissues. Despite seeing no yield effects from inoculation, we found that across treatments percent root length colonized by AMF was positively correlated with plant tissue P, Cu, and Zn concentration. Underlying these broad benefits, seeds from plants inoculated with three AMF species, *Claroideoglomus claroideum* (+15%), *Funneliformis mosseae* (+13%), and *Gigaspora rosea* (+11%) had higher P concentration than non‐mycorrhizal plants. *C. claroideum* also increased seed potassium (K) and copper (Cu), as well as leaf aluminum (Al) concentration making it a promising candidate to further test the benefit of individual AMF species on black bean growth in field trials.

## Graphical abstract

Mycorrhizal fungi have proven to be useful biofertilizers for a range of crops. Here, we show that several individual species of mycorrhizal fungi are promising candidates for improving the nutritional quality of black beans (*Phaseolus vulgaris*).

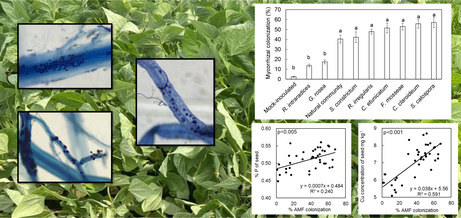

## 1 INTRODUCTION

Public concern over the negative environmental impacts of the overuse of agrichemicals has driven an increased interest in sustainable agriculture and a demand for organically grown food (Reganold & Wachter, 2016; Willer et al., 2021). Therefore, there is a need to develop technologies that enhance crop nutrient uptake to counter reductions in mineral nutrient inputs when adhering to organic practices. In response to this demand, research on the use of arbuscular mycorrhizal fungi (AMF) as biofertilizers in crop production has gained momentum (Berruti et al., 2016; Igiehon & Babalola, 2017; Jerbi et al., 2022; Madawala, 2021; Sadhana, 2014). AMF grow in association with the roots of >80% of land plants and trade soil nutrients and water for photosynthetically derived carbon (C) and additionally bolster plants' resistance to pathogens and other environmental stressors (Read & Perez‐Moreno, 2003). While AMF are naturally present in all agricultural soils, agricultural management practices which increase AMF abundance have the potential to bolster the yield and nutritional quality of a variety of crops, including common bean (*Phaseolus vulgaris* black turtle: herein black bean).

Much of the past research on using AMF to supplement or replace mineral fertilizers has focused on inoculating plants with on‐farm produced mixed species AMF inocula which have been propagated from the natural AMF communities in field soils or on commercial products containing the single AMF species *Rhizophagus irregularis* (Douds et al., 2005, 2010). The mixed‐species inoculation method has been shown to increase the yield of a range of fruits and vegetables such as potatoes, sweet potatoes, peppers, and eggplant by up to 15% (Douds et al., 2014, 2015, 2017; Douds & Reider, 2003). The commercial *Rhizophagus irregularis* products have shown positive, but less consistent yield results, which are often contributed to inconsistencies in viable AMF spores in products that are stored and shipped over long distances (Corkidi et al., 2004; Faye et al., 2013; Salomon et al., 2022). In addition to the success of AMF inoculation in bolstering yield, additional benefits of AMF inoculation on plant nutritional quality include increases in mineral nutrient uptake (Chandrasekaran, 2020; Clark & Zeto, 2000), anthocyanin concentration (Avio et al., 2017; Baslam et al., 2011; Chiomento et al., 2019; Nacoon et al., 2023; Tisarum et al., 2019), and overall antioxidant activity across a range of vegetables and fruits (Hart et al., 2015; Mollavali et al., 2016; Toussaint et al., 2007). Increases in the mineral nutrient concentration of foods can improve public health by preventing nutrient deficiency which affects over 2 billion people worldwide (Tulchinsky, 2010). Increased anthocyanin concentration of foods can also have positive effects on public health by preventing diseases brought on by oxidative stress (Gonçalves et al., 2021).

Past work examining plant responses to inoculation with individual AMF species suggests that the impact of AMF on nutrient uptake varies by fungal and host species wherein the relationship lies on a spectrum of mutualism to fungal parasitism (Carrara & Heller, 2022; Klironomos, 2003; Mensah et al., 2015; Munkvold et al., 2004; Smith et al., 2004). This observed variability in the benefits of mycorrhizal inoculation on host plants is likely due to a range in the plant C costs of nutrient return across AMF species. The most cost‐effective AMF species, or the AMF species with the highest nutrient acquisition efficiency, provides the greatest benefit to plant fitness (Carrara & Heller, 2022; Carrara et al., 2023; Carrara et al., 2023; Kiers et al., 2011; Van't Padje et al., 2021; Werner & Kiers, 2015). As such, trials that assess plant responses to inoculation with a variety of individual AMF species are a promising avenue toward developing host‐targeted inocula that can enhance nutrient uptake and crop yield to the greatest extent.

Common beans (*Phaseolus vulgaris*) account for half of the grain legumes consumed worldwide and are a significant protein source in Latin America, Africa, and parts of Asia (Broughton et al., 2003; McClean et al., 2011). As a staple crop in many countries in the developing world, many of which suffer from both food shortage and human nutrient deficiency, common bean is a prime candidate for the use of mycorrhizal fungi as a tool to improve yield and nutritional quality. In this study, we inoculated black beans with eight different monospecific AMF accessions (Table 1) and one mixed‐species AMF community indigenous to organically managed field plots at the Rodale Institute, Kutztown, PA, USA in a pot trial to examine the extent to which mycorrhizal species impacted plant tissue mineral nutrient concentration, anthocyanin concentration, and yield. To do this, we measured percent of the root length colonized by AMF within each treatment and compared this to measurements of the concentration of anthocyanins and the 12 most abundant macro‐ and micro‐nutrients in black bean leaves and seeds at harvest.

**TABLE 1:** Mycorrhizal accessions used to create AMF treatment inocula.

AMF species	Accession ID	Source	Site of origin
*Claroideoglomus claroideum*	PA104A	INVAM	Kutztown, PA, USA
*Claroideoglomus etunicatum*	PA127B	INVAM	Pennsylvania, USA
*Funneliformis mosseae*	PA127A	INVAM	Pennsylvania, USA
*Gigaspora rosea*	FL105	INVAM	Florida, USA
*Scutellospora calospora*	PA103A	INVAM	Pennsylvania, USA
*Septoglomus constrictum*	LGEO	ERRC	Kutztown, PA, USA
*Rhizophagus intraradices*	QE102	INVAM	Quebec, Canada
*Rhizophagus irregularis*	ON205B	INVAM	Ontario, Canada
Natural community	N/A	Organic vegetable soil	Kutztown, PA, USA

*Note*: INVAM is the International collection of (Vesicular) Arbuscular Mycorrhizae housed at West Virginia University, Morgantown, WV, USA. ERRC is the USDA Agricultural Research Service, Eastern Regional Research Center, Wyndmoor, PA, USA.

## 2 METHODS

### 2.1 Trap culture production of AMF inocula

Individual AMF species accessions were sourced from the International Collection of Vesicular Arbuscular Mycorrhizae (INVAM) housed at West Virginia University, Morgantown WV, USA, except for *Septoglomus constrictum* which was sourced from the AMF collection of the United States Department of Agriculture, Agricultural Research Service, Eastern Regional Research Center, Wyndmoor, PA, USA (Table 1). We chose these AMF species due to their prevalence in agricultural field soils.

AMF were maintained in the greenhouse in Bahia grass (*Paspalum notatum* Flügge) pot cultures grown in a 1:0.75:1:0.75 v/v combination of sterilized sand, soil, vermiculite, and turface as in Douds et al. (2010). Soil for the Bahia grass pot cultures was taken from organically managed agricultural plots at the Rodale Institute, Kutztown PA, USA. A mixed natural AMF population was propagated by preparing a 1:10 dilution of field soil also from organically managed agricultural plots at the Rodale Institute. After 1 year of growth, inocula were prepared by removing the aboveground tissue of the Bahia grass prior to cutting the roots into ~1 cm lengths and reincorporating them into the media. We also prepared “mock” inocula to account for any additional nutrients added with the substrate when inoculating the black beans by growing Bahia grass without mycorrhizal fungi and using this substrate to “mock inoculate” our control black bean plants. Molecular analysis by multiplex qPCR (Heller & Carrara, 2022) was used to confirm the monospecific inocula were free from contamination by other AMF, and indicated that the mixed species natural community inoculum contained *C.etunicatum*, *C. claroideum*, *F. mosseae*, *R. intraradices*, and *R. irregularis*.

### 2.2 Planting

Black bean seeds were sourced from Seneca Grain & Bean (Penn Yan, NY) and grown in 2 L Treepots (Stuewe and Sons, Tangent, OR) in a mix of 2:2:1 sterilized field soil: peat moss:turface. Like the Bahia grass pot cultures, the soil used in the black bean potting mix was sourced from organically managed vegetable plots at the Rodale Institute and soil properties were analyzed by the Pennsylvania State University Agricultural Analytical Laboratory, State College, PA after sterilization via autoclave (Table S1). We used sterilized soil to minimize competition and any interactive effects between the natural microbial community present in the field soil and the mycorrhizal fungi used in the inocula. AMF inocula (spore‐laden Bahia grass potting substrate with reincorporated colonized root fragments) were added into the black bean growth media at a concentration of 2000 spores/pot. Seeds were pre‐inoculated with Guard‐N rhizobium legume seed inoculant (Verdesian Life Sciences, Cary, NC) according to the manufacturer's recommendation prior to sowing. In each pot, four black bean seeds were sown on June 03, 2021 and grown outdoors on raised benches arranged randomly in unobstructed sunlight. The AMF species treatments were *S. constrictum, F. mosseae, R. irregularis, C. claroideum, C. etunicatum, S. calospora, G. rosea, R. intraradices*, and the natural community produced from organically managed field soil from the Rodale Institute (Table 1). For each mycorrhizal treatment, there were 12 pots for a total of 48 individual plants. Plants were watered daily and fertilized once weekly with Hoagland's solution modified to contain 0.1X phosphorus (P; Hoagland & Arnon, 1938). Although the soil used in this experiment was sourced from an agricultural field, and therefore does not have low phosphate availability (Table 1), low P concentration fertilizer is commonly used in mycorrhizal pot inoculation studies to elicit P limitation to promote root‐spore signaling and root colonization (Douds & Schenck, 1990).

### 2.3 Harvest and tissue analysis

Black bean leaves were harvested upon flowering on August 08, 2021. The newest, fully mature leaf from each plant from three pots within each treatment was composited for mineral nutrient analysis (12 plants per composite sample, 4 replications per treatment). After harvest, leaves were dried at 55°C for 1 week and ground to powder in a tube mill prior to mineral nutrient analysis.

Black bean seeds were harvested on August 31, 2021 and, similarly to leaves, all seeds from three pots were composited within treatment for mineral nutrient analysis (12 plants per composite sample, 4 replications per treatment). After collection, pods were dried at 55°C for 1 week. Seed yield was determined after threshing and seeds were then ground to powder in a tube mill prior to mineral nutrient and anthocyanin analysis. Root samples were composited in the same way as leaves and seeds upon harvest for determination of AMF root colonization.

Dried and ground leaf and seed materials were sent to Penn State University Agricultural Analytical Laboratory, State College, PA. Tissues were analyzed for the concentration of phosphorus (P), potassium (K), calcium (Ca), magnesium (Mg), and sulfur (S), iron (Fe), copper (Cu), boron (B), aluminum (Al), zinc (Z), and sodium (Na) by nitric acid (HNO_3_) and peroxide (H_2_O_2_) digestion followed by inductively coupled plasma–optical emission spectrometry analysis (Huang & Schulte, 1985). Anthocyanin analysis was conducted at Purdue University, West Lafayette Indiana, on dried and ground seed material by the pH differential method (Wrolstad, 1993) modified as in Madiwale et al., 2011.

### 2.4 Determination of Mycorrhizal colonization

Percent of root length colonized by AMF was determined by trypan blue staining followed by the root intercept scoring method. First, media was removed by gently washing the roots in water. The roots were then soaked in 10% potassium hydroxide (KOH) for ~96 h and subsequently brought to a boil to remove cell contents. The cleared roots were rinsed twice in tap water, soaked in 1.5% alkaline peroxide solution room temperature for 20 min to remove pigments, then acidified in 1% hydrochloric acid (HCl) and stained by bringing them to a boil in 0.5% trypan blue solution (Phillips & Hayman, 1970). Percent of the root length colonized was then determined by the grid‐intersect method (Giovannetti & Mosse, 1980).

### 2.5 Statistical analysis

To determine significant differences in tissue nutrient concentrations between AMF treatments, we ran a one‐way analysis of variance (ANOVA) with AMF treatment as the main factor on leaf and seed mineral nutrient concentrations, anthocyanin concentration, and yield separately. After determining significance of ANOVA (*p* < .05) we used Tukey–Kramer HSD to compare mean nutrient concentrations between AMF treatments. To determine significance in the correlation between percent colonization and leaf and seed nutrient concentrations we performed Pearson's product–moment tests. All statistical analyses were performed in R version 4.2.1 (R Team, 2022).

## 3 RESULTS

### 3.1 Mycorrhizal colonization and seed yield

**FIGURE 1:**
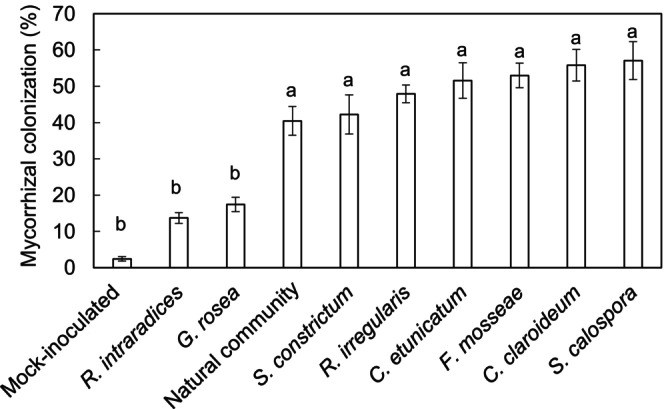
Percent mycorrhizal colonization of black bean roots varied by AMF species. Values presented are means accompanied by standard error (*n* = 4). Letters indicate significant differences between treatments as determined by Tukey–Kramer HSD following significant ANOVA (*p* < .05).

Individual AMF species treatments exhibited a range of root‐colonization efficiency. The AMF species that had the highest root colonization were *S. calospora* and *C. claroideum* with 57% and 56% of root length colonized, respectively (Figure 1). The AMF species with the lowest percent colonization were *G. rosea* and *R. intraradices* at 17% and 14% of root length colonized respectively (Figure 1). There was a small amount of AMF colonization in the mock‐inoculated plants at final harvest (<3%) which was likely due to environmental transfer (i.e., wind, insects) of spores to the surface of the pots as the result of being grown outside. In addition to mycorrhizal fungi, the roots contained functional rhizobia nodules in every treatment. There were no significant seed yield differences between any AMF‐inoculated and mock‐inoculated treatments (Table S2).

### 3.2 Leaf and seed nutrient concentration

Seed P concentration was higher in plants inoculated with *C. claroideum* (+15%), *F. mosseae* (+13%), and *G. rosea* (+11%) compared to mock‐inoculated plants (Table 2). Seed K concentration was 8% and 5% higher in plants inoculated with *C. claroideum* and *C. etunicatum*, respectively. Seed Mg concentration was 8% lower in *S. constrictum* inoculated plants compared to mock‐inoculated plants (Table 2). Seed Fe concentration was 15% and 20% lower in *S. constrictum* and *R. intraradices* inoculated plants compared to controls. Cu seed concentration was higher in plants inoculated with *C. claroideum* (38%), *R. irregularis* (27%), *C. etunicatum* (24%), and *F. mosseae* (23%) than mock inoculated plants. Seed B concentration was about 19% lower in plants inoculated with *G. rosea* and the natural community than mock‐inoculated plants. Seed S concentration was 17% higher in plants inoculated with *C. claroideum*. Finally, seed Na concentration was about 20% lower in 7 of 9 of the AMF treatments compared to mock‐inoculated plants (Table S3).

**TABLE 2:** Concentration of mineral macronutrients in black bean seeds.

AMF treatment	N (%)	P (%)	K (%)	Ca (%)	Mg (%)	Anthocyanin, mg 100 g^−1^
Mock‐inoculated	3.43 ± 0.09	0.47 ± 0.01^d^	1.34 ± 0.01^bcd^	0.11 ± 0.00	0.18 ± 0.00^ab^	29.6 ± 2.5^bc^
*C. claroideum*	3.75 ± 0.12	**0.55 ± 0.01** ^**a**^	**1.44 ± 0.02** ^**a**^	0.09 ± 0.00	0.19 ± 0.00^ab^	29.3 ± 1.7^bc^
*C. etunicatum*	3.94 ± 0.04	0.52 ± 0.01^abcd^	**1.41 ± 0.02** ^**ab**^	0.09 ± 0.01	0.18 ± 0.00^abc^	35.8 ± 0.9^ab^
*F. mosseae*	3.61 ± 0.14	**0.53 ± 0.01** ^**ab**^	1.40 ± 0.01^ab^	0.09 ± 0.01	0.18 ± 0.00^abc^	**38.9 ± 0.2** ^**a**^
*G. rosea*	3.75 ± 0.05	**0.53 ± 0.01** ^**abc**^	1.38 ± 0.01^abc^	0.10 ± 0.01	0.19 ± 0.00^a^	28.3 ± 2.1^bc^
*S. calospora*	3.67 ± 0.09	0.52 ± 0.01^abcd^	1.31 ± 0.02^cd^	0.09 ± 0.01	0.18 ± 0.00^abc^	29.0 ± 1.4^bc^
*S. constrictum*	3.46 ± 0.20	0.51 ± 0.01^abcd^	1.30 ± 0.02^d^	0.10 ± 0.01	**0.17 ± 0.00** ^**c**^	**26.6 ± 1.6** ^**c**^
*R. intraradices*	3.63 ± 0.13	0.48 ± 0.00^cd^	1.36 ± 0.02^abcd^	0.10 ± 0.01	0.18 ± 0.00^bc^	35.5 ± 3.0^ab^
*R. irregularis*	3.54 ± 0.14	0.51 ± 0.00^abcd^	1.37 ± 0.02^abcd^	0.10 ± 0.01	0.18 ± 0.00^bc^	**27.1 ± 1.0** ^**c**^
Natural community	3.51 ± 0.06	0.50 ± 0.01^bcd^	1.33 ± 0.02^cd^	0.09 ± 0.01	0.18 ± 0.00^bc^	**25.1 ± 1.0** ^**c**^

*Note*: Mineral nutrient values are % tissue dry mass means ± standard error. Anthocyanin is expressed in mg 100 g^−1^ of dry tissue. Letters indicate significant differences between treatments and bold values highlight significant differences between treatment and mock‐inoculated (control) plants as determined by Tukey–Kramer HSD following significant ANOVA (*p* < .05). No letters indicate insignificant ANOVA.

There were fewer differences among leaf nutrient concentrations between AMF‐inoculated and mock‐inoculated plants. Leaf K concentration was 26% and 21% higher in *S. constrictum* and *F. mosseae* inoculated plants than in mock‐inoculated plants (Table 3). Leaf B concentration was about 15% lower in plants inoculated with *F. mosseae*, *G. rosea*, *R. intraradices*, and *R. irregularis* compared to mock‐inoculated plants and leaf Al concentration was 120% higher in plants inoculated with *C. claroideum* (Table S4).

**TABLE 3:** Concentration of mineral macronutrients in black bean leaves.

AMF treatment	N (%)	P (%)	K (%)	Ca (%)	Mg (%)
Mock‐inoculated	4.45 ± 0.24	0.40 ± 0.03	2.33 ± 0.16^c^	1.56 ± 0.08	0.43 ± 0.01
*C. claroideum*	4.79 ± 0.18	0.41 ± 0.03	2.46 ± 0.05^bc^	1.32 ± 0.10	0.42 ± 0.01
*C. etunicatum*	5.05 ± 0.07	0.45 ± 0.02	2.3 ± 0.05^abc^	1.34 ± 0.06	0.41 ± 0.01
*F. mosseae*	4.92 ± 0.22	0.47 ± 0.04	**2.83 ± 0.09** ^**ab**^	1.18 ± 0.12	0.36 ± 0.01
*G. rosea*	5.00 ± 0.16	0.46 ± 0.02	2.72 ± 0.11^abc^	1.32 ± 0.06	0.40 ± 0.02
*S. calospora*	5.09 ± 0.20	0.45 ± 0.03	2.58 ± 0.02^abc^	1.34 ± 0.01	0.43 ± 0.01
*S. constrictum*	4.90 ± 0.23	0.48 ± 0.03	**2.95 ± 0.14** ^**a**^	1.16 ± 0.08	0.40 ± 0.01
*R. intraradices*	4.9 ± 0.06	0.45 ± 0.03	2.60 ± 0.10^abc^	1.35 ± 0.14	0.42 ± 0.03
*R. irregularis*	4.60 ± 0.21	0.39 ± 0.02	2.53 ± 0.05^abc^	1.43 ± 0.06	0.44 ± 0.01
Natural community	4.70 ± 0.09	0.40 ± 0.02	2.38 ± 0.08^bc^	1.27 ± 0.04	0.43 ± 0.01

*Note*: Values are means ± standard error. Values are % tissue dry mass means ± standard error. Letters indicate significant differences between treatments and bold values highlight significant differences between treatment and mock‐inoculated (control) plants as determined by Tukey–Kramer HSD following significant ANOVA (*p* < .05). No letters indicate non‐significant ANOVA.

**FIGURE 2:**
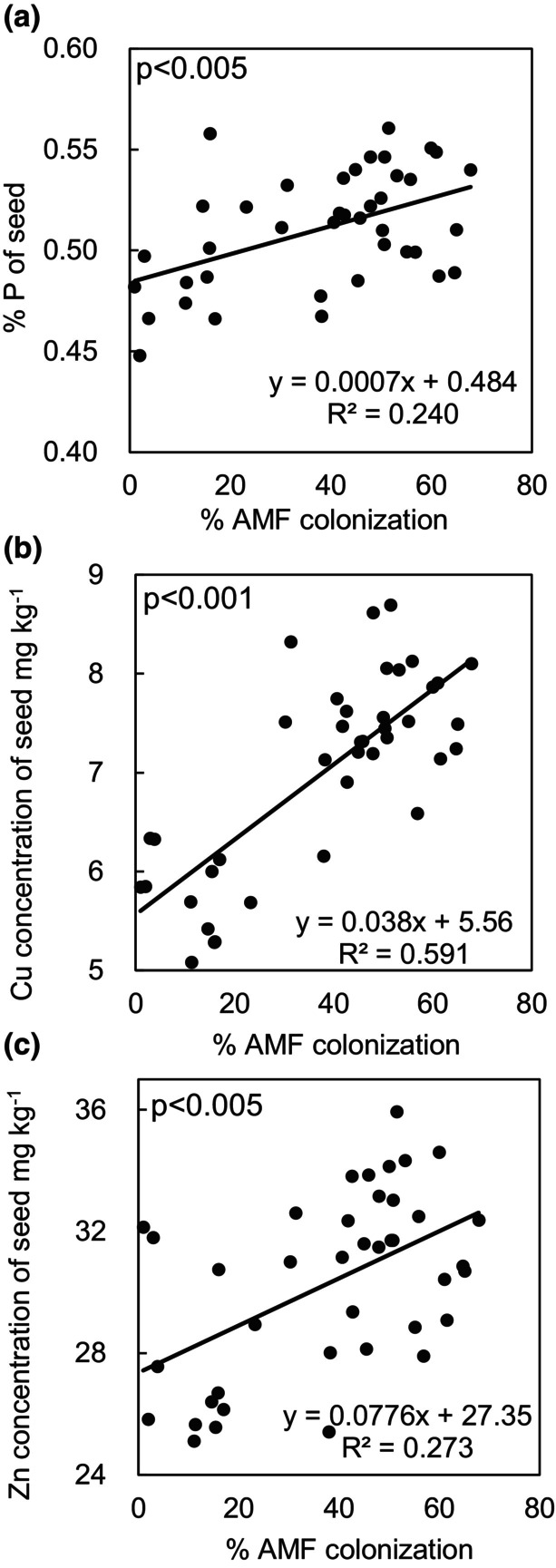
Percent mycorrhizal root colonization was positively correlated with seed tissue concentrations of P (a), Cu (b), and Zn (c) of black beans across all AMF treatments. *p* values are from Pearson‐moment correlation tests.

**FIGURE 3:**
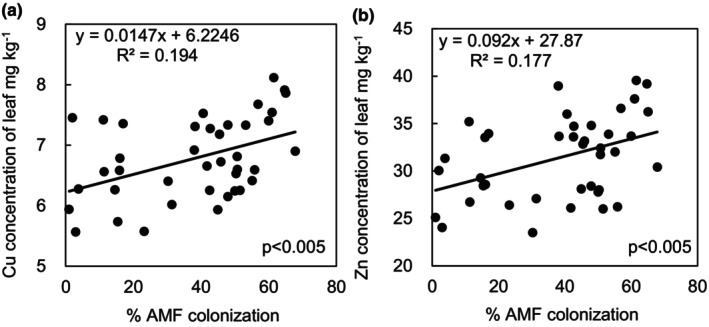
Percent mycorrhizal root colonization was positively correlated with leaf tissue concentrations of Cu (a) and Zn (b) of black beans across all treatments. *p* values are from Pearson‐moment correlation tests.

In bean seeds, across all treatments, percent root length colonized was positively correlated with P, Cu, and Zn concentration (Figure 2). In leaves, across all treatments, percent root length colonized was positively correlated with Cu and Zn concentration (Figure 3).

### 3.3 Anthocyanin concentration

There was a mixed response of seed anthocyanin concentration to inoculation across AMF treatments compared to the mock‐ inoculated treatment. One AMF species, *F. mosseae*, increased total seed anthocyanin concentration by 32%, while three treatments (*R. irregularis*, natural community, and *S. constrictum*) reduced anthocyanin concentration by 8%–15% compared to mock‐inoculated plants (Table 2).

## 4 DISCUSSION

Increased demand for organic produce has bolstered research on the use of AMF as biofertilizers to supplement or replace agrichemicals (Berruti et al., 2016; Igiehon & Babalola, 2017; Jerbi et al., 2022; Madawala, 2021; Sadhana, 2014). Here we show that several single‐species AMF inocula have a positive impact on black bean mineral nutrient and anthocyanin concentration. We posit that the range of AMF species effectiveness at enhancing plant mineral nutrient concentration may be driven by differences between the plant‐C cost of P return among AMF species. This finding provides a basis for undertaking additional trials that assess the effectiveness of individual AMF species at enhancing host nutritional status across a variety of crops (Kiers et al., 2011; Mensah et al., 2015; Werner & Kiers, 2015). An additional explanation for plant mineral nutrient concentration differences between AMF treatments are differences in the growth rate and nutrient acquisition efficiency between mycorrhizal taxa, independent of host C allocation (Corrêa et al., 2015).

The most commonly reported benefit of mycorrhizal inoculation toward plant nutrition is the enhancement of P uptake (Adeyemi et al., 2021; Cardoso et al., 2006; Carrara & Heller, 2022; Conversa et al., 2013; Karandashov & Bucher, 2005; Khaliq & Sanders, 2000; Ortas et al., 1996; Thioub et al., 2019). In P limited systems plants rely on mycorrhizal hyphae to scavenge immobile P from soil aggregates and micropores that are inaccessible to roots (Smith et al., 2011). We found that across all treatments, percent mycorrhizal colonization was positively correlated with seed P concentration which indicated that plants allocated C toward P acquisition through the mycorrhizal pathway (Figure 2a). Correlation between percent root length colonized and plant P concentration agrees with our previous pot trials in corn (*Zea Mays*) and squash (*Curcurbita moschata*) (Carrara & Heller, 2022) as well as many other studies as reviewed by Treseder (2013). However, while percent colonization and seed P concentration were positively correlated across all treatments, only three mycorrhizal species (*C. claroideum*, *F. mosseae*, and *G. rosea*) had significantly higher seed P concentration than mock inoculated seeds (Table 2). Further, despite resulting in an increase in seed P concentration, *G. rosea* colonized roots had a relatively low level of root colonization (17%; Figure 1) and *S. calospora*, which had the highest level of root colonization (57%), did not increase seed P concentration when compared to mock‐inoculated seeds (Figure 1, Table 2). It is possible that this result was driven by differences in the plant‐C cost of P transfer between AMF species. Alternatively, faster growing AMF species or species with a high efficiency for acquiring soluble nutrients (i.e., high surface area, fast growing hyphae) may have driven the higher rates of host mineral nutrient transfer seen here.

While we found evidence that some AMF species enhanced P uptake and an overall positive correlation between percent AMF colonization and seed P concentration, no AMF species impacted seed yield (Table S2). One explanation for this may be that the plants were root‐bound and thus space‐limited by harvest which negated any initial benefit of mycorrhizal colonization and limited adequate nutrient uptake during pod‐filling. Additionally, it is possible that plant growth was limited by another nutrient such as N, although it is unlikely as these plants were co‐inoculated with rhizobia, an important root‐symbiont of legumes which fix atmospheric N and provides an important N source for the host (Peoples et al., 1995). As past research has shown a positive yield response in leguminous hosts inoculated with a combination of AMF and nitrogen‐fixing symbionts, it is more likely that the lack of yield response in this study was the result of container size (Larimer et al., 2010; Mortimer et al., 2008; Razakatiana et al., 2020). We also found that while seed P concentration was correlated with mycorrhizal colonization, leaf P was not. It is possible that this was due to different sampling times (i.e., leaves sampled at flowering, seeds sampled at maturity) or differences in nutrient allocation within the plant. Future studies would benefit by sampling tissues at multiple timepoints to inform growers of optimal times for testing the impact of mycorrhizal inoculation through leaf sampling before harvest.

In addition to enhanced P uptake, inoculation with 4 AMF species (*C. claroideum, C. etunicatum, F. mosseae, R. irregularis*) resulted in increased seed Cu concentration (Table S3). Further, percent AMF colonization across treatments was positively correlated with Cu concentration in both seed and leaf tissue (Figures 2b and 3a). This is in line with a meta‐analysis of over 200 AMF field inoculation trials that demonstrated that AMF are an important mediator of Cu uptake across a wide variety of host plant species (Lehmann & Rillig, 2015). Root access to Cu is limited due to its affinity to form complexes with a range of organic and inorganic compounds in soil rendering it immobile (McLaren et al., 1983); thus, this result suggests that plants benefited from the enhanced soil scavenging capacity of mycorrhizal mycelial networks. Similarly, across treatments, percent AMF colonization was also positively correlated with both seed and leaf Zn concentration (Figures 2c and 3b). Like Cu, AMF mediation of Zn uptake has been demonstrated in a variety of hosts including grapes, acai, switchgrass, wheat, barley, pepper, and maize (Chu, 1999; Clark, 2002; Coccina et al., 2019; Javaid, 2009; Karagiannidis & Nikolaou, 1999; Ortas et al., 2011; Ryan & Angus, 2003; Saboor et al., 2021; Schreiner, 2007). Increased Zn concentration of beans may have positive implications for human nutrition as about 17% of the world's population are at risk of Zn deficiency which results in reduced immune function and stunted growth among many other physiological complications (Belay et al., 2021; Wessells & Brown, 2012). Finally, while the mechanisms that influence the impact of mycorrhizal fungi on anthocyanin plant concentration are unclear, inoculation with *F. mosseae* enhanced black bean anthocyanin concentration by 42%, a result in line with mycorrhizal inoculation studies of strawberry and lettuce (Avio et al., 2017; Chiomento et al., 2019). Increased anthocyanin concentrations of crops can benefit human nutrition due to their antioxidant capacity and anti‐inflammatory effects (Pojer et al., 2013).

Although the natural community AMF treatment likely contained most of the individual AMF species tested here, none of the increases in tissue mineral nutrient or anthocyanin concentrations brought on by inoculation with individual AMF species occurred in the natural community mixed species treatment. This result agrees with a recent study in our laboratory which examined the impact of inoculation with several individual AMF species as well as a mixed AMF community on corn and squash nutrient uptake and found that single accession AMF treatments benefitted mineral nutrient uptake to a greater extent than mixed communities (Carrara & Heller, 2022). It is possible that this phenomenon is the result of competition for access to root surface area between AMF species that have a lower C cost of nutrient acquisition with less efficient AMF species, but this hypothesis requires further testing which may employ the strategy of inoculating hosts with combinations of individual AMF species with equal spore densities. Additionally, we previously reported that seven of these AMF treatments, including the mixed‐species treatment, increased black bean uptake of the compound ergothioneine from soils, an antioxidant synthesized by fungi that is gaining traction as a potential human health vitamin (Beelman et al., 2020, 2022; Carrara, Lehotay, et al., 2023; Carrara, Reddivari, et al., 2023). Therefore, future studies would benefit by measuring additional nutritional quality indicators in controlled AMF studies.

While there are limitations to this study, we argue that determining the extent to which a range of individual AMF species enhance the nutritional quality of black beans is an important step in the development of host‐targeted biofertilizers that will be effective in the field setting. One obstacle in testing the relationship between individual species of AMF and host plants is that the soil must be sterilized (in our case via autoclave) to eliminate the extant microbial community. This process hides the impact of the natural soil microbial community on the viability of the added AMF and also results in the release of plant‐available nutrients that may contribute to plant growth (Dietrich et al., 2020; Hendriks et al., 2013). However, because all of the AMF species were inoculated at the same rate and exposed to the same growth conditions in this study, we posit that these results are useful in informing field trials aimed at the development of AMF biofertilizers.

### 4.1 Conclusion

Overall, we found differences in the impact of AMF inoculation on black bean tissue nutrient concentration across eight individual AMF species and one mixed natural AMF community. At the broad scale, these results show that efforts to increase total mycorrhizal colonization of black beans regardless of species will likely have positive implications for P, Cu, and Zn uptake overall, but inoculation with individual species including *C. claroideum*, *F. mosseae*, and *G. rosea* may have even greater benefits to P uptake than using a mixed community inoculum. Additionally, *C. claroideum* inoculated plants also had higher seed K, Cu, and S concentrations and higher leaf Al concentrations which could provide additional benefits to black bean health in acidic soils. As such, *C. claroideum* appears to be a promising individual AMF species for increasing the nutritional quality of black beans. Further testing should focus on inoculating black beans with the most beneficial AMF species described here in the field setting to determine if the apparent benefits of inoculation are not muted by competition with the natural AMF community.

## CONFLICT OF INTEREST STATEMENT

The authors declare no conflicts of interest.

## Supporting information


Table S1.
Table S2.
Table S3.
Table S4.

## Data Availability

The original contributions presented in the study are included in the article and Supplementary Material. Further inquiries can be directed to the corresponding author.
