# Supplementary material for: Inoculation of black turtle beans (Phaseolus vulgaris) with mycorrhizal fungi increases the nutritional quality of seeds
Source: Plant Environ Interact. 2023 Nov 15;5(1):e10128. doi: 10.1002/pei3.10128 (PMC10840373; doi:10.1002/pei3.10128)
Supplement: Supplementary file 1 — Table S1. Table S2. Table S3. Table S4. [file PEI3-5-e10128-s001.docx]

**Supplemental information**

**Inoculation of black turtle beans (*Phaseolus vulgaris*) with mycorrhizal fungi increases the nutritional quality of seeds**

**Authors**

Joseph E. Carrara^1^, Lavanya Reddivari^2^, Wade P. Heller^1^

**Affiliations**

^1^USDA Agricultural Research Service, Eastern Regional Research Center, Wyndmoor, Pennsylvania, USA.

^2^Department of Food Science, Purdue University, West Lafayette, IN, USA

**Table S1.** Soil properties of sterilized soil used in the substrate mix.

| **Unit** | **Property** | **Value** |
| --- | --- | --- |
|  | **% Nitrogen** | 0.9 ± 0.01 |
|  | **Soil pH** | 7.5 ± 0.1 |
| **Soil Nutrient Level**  **(kg ha^-1^)** | **Phosphate (P2O5)** | 1421.6 ± 24.8 |
|  | **Potash (K2O)** | 740.1 ± 14.9 |
|  | **Magnesium (MgO)** | 1191.0 ± 36.39 |
|  | **Calcium (CaO)** | 7563.2 ± 152.8 |
|  | **Phosphorus (P)** | 620.9 ± 10.9 |
| **Exchangeable Cations**  **(meq 100g^-1^)** | **K** | 0.9 ± 0.00 |
|  | **Mg** | 3.0 ± 0.1 |
|  | **Ca** | 15.3 ± 0.3 |
| **% Saturation of CEC** | **K** | 4.7 ± 0.1 |
|  | **Mg** | 15.8 ± 0.4 |
|  | **Ca** | 79.4 ± 0.4 |
|  | **% Organic Matter** | 14.9 ± 0.4 |
|  | **Nitrate-N ppm** | 110.7 ± 3.1 |
|  | **NH4-N ppm** | 69.4 ± 2.3 |
|  | **CEC** | 18.9 ± 0.1 |

**Table S2.** Dry mass of black bean seed yield did not vary between arbuscular mycorrhizal fungi (AMF) treatments. Numbers are average values (composite of 12 plants) presented with standard error (n=4).

| **AMF Treatment** | **seed yield dry mass (g)** | | |
| --- | --- | --- | --- |
| Mock | 25.4 | ± | 2.2 |
| *R. intraradices* | 30.4 | ± | 2.4 |
| Natural community | 29.8 | ± | 2.4 |
| *S. calospora* | 28.3 | ± | 2.9 |
| *G. rosea* | 28.1 | ± | 2.0 |
| *S. constrictum* | 27.8 | ± | 1.9 |
| *R. irregularis* | 27.7 | ± | 1.4 |
| *C. etunicatum* | 25.7 | ± | 1.2 |
| *F. mosseae* | 25.7 | ± | 1.2 |
| *C. claroideum* | 18.9 | ± | 0.4 |

**Table S3.** Mineral micronutrient concentration of black bean seeds. S is expressed in %, all other minerals are expressed in mg kg^-1^ and values are ± standard error. Letters indicate significant differences between treatments and bold values highlight significant differences between treatment and mock-inoculated (control) plants as determined by Tukey-Kramer HSD following significant ANOVA (p<0.05). No letters indicate non-significant ANOVA.

| **AMF Treatment** | **S (%)** | **Fe (mg kg^-1^)** | **Cu (mg kg^-1^)** | **B (mg kg^-1^)** | **Al (mg kg^-1^)** | **Zn (mg kg^-1^)** | **Na (mg kg^-1^)** |
| --- | --- | --- | --- | --- | --- | --- | --- |
| **Mock-inoculated** | 0.22 ± 0.01^cde^ | 82.78 ± 4.98^a^ | 6.09 ± 0.14^cde^ | 12.18 ± 0.38^a^ | 1.25 ± 0.00 | 29.33 ± 1.56^ab^ | 7.82 ± 0.31^a^ |
| ***C. claroideum*** | **0.26 ± 0.00^a^** | 74.78 ± 1.29^abc^ | **8.38 ± 0.16^a^** | 10.65 ± 0.06^ab^ | 1.24 ± 0.00 | 33.49 ± 0.83^a^ | **6.18 ± 0.02^b^** |
| ***C. etunicatum*** | **0.25 ± 0.00^ab^** | 79.18 ± 1.03^ab^ | **7.55 ± 0.18^ab^** | 10.78 ± 0.37^ab^ | 1.24 ± 0.01 | 33.21 ± 0.79^a^ | **6.20 ± 0.03^b^** |
| ***F. mosseae*** | 0.23 ± 0.00^bcd^ | 75.79 ± 1.45^abc^ | **7.50 ± 0.15^ab^** | 10.20 ± 0.34^ab^ | 1.24 ± 0.01 | 30.97 ± 0.89^a^ | **6.36 ± 0.17^b^** |
| ***G. rosea*** | 0.23 ± 0.00^bcde^ | 73.59 ± 2.00^abc^ | 5.42 ± 0.09^e^ | **9.88 ± 0.34^b^** | 1.26 ± 0.01 | 28.19 ± 1.02^b^ | **6.28 ± 0.04^b^** |
| ***S. calospora*** | 0.22 ± 0.00^bcde^ | 74.37 ± 2.21^abc^ | 7.49 ± 0.15^abc^ | 11.34 ± 0.95^ab^ | 1.24 ± 0.01 | 31.68 ± 1.18^a^ | 7.09 ± 0.57^ab^ |
| ***S. constrictum*** | 0.21 ± 0.00^e^ | **69.99 ± 0.77^bc^** | 6.99 ± 0.47^bcd^ | 10.85 ± 0.26^ab^ | 1.25 ± 0.01 | 28.81 ± 1.50^ab^ | 7.39 ± 0.25^ab^ |
| ***R. intraradices*** | 0.21 ± 0.01^de^ | **66.13 ± 1.93^c^** | 5.72 ± 0.23^de^ | 10.49 ± 0.35^ab^ | 1.25 ± 0.01 | 25.62 ± 0.21^b^ | **6.36 ± 0.11^b^** |
| ***R. irregularis*** | 0.24 ± 0.00^abc^ | 72.80 ± 0.73^abc^ | **7.70 ± 0.13^ab^** | 10.93 ± 0.31^ab^ | 1.23 ± 0.00 | 32.17 ± 0.67^a^ | **6.17 ± 0.02^b^** |
| **Natural community** | 0.23 ± 0.00^bcd^ | 71.24 ± 2.04^abc^ | 7.28 ± 0.08^abc^ | **9.98 ± 0.19^b^** | 1.25 ± 0.01 | 29.66 ± 0.92^ab^ | **6.31 ± 0.06^b^** |

**Table S4.** Mineral micronutrient concentration of black bean leaves. S is expressed in %, all other minerals are expressed in mg kg^-1^ and values are ± standard error. Letters indicate significant differences between treatments and bold values highlight significant differences between treatment and mock-inoculated (control) plants as determined by Tukey-Kramer HSD following significant ANOVA (p<0.05). No letters indicate insignificant ANOVA.

| **AMF Treatment** | **S (%)** | **Fe (mg kg^-1^)** | **Cu (mg kg^-1^)** | **B (mg kg^-1^)** | **Al (mg kg^-1^)** | **Zn (mg kg^-1^)** | **Na (mg kg^-1^)** |
| --- | --- | --- | --- | --- | --- | --- | --- |
| **Mock-inoculated** | 0.26 ± 0.01 | 104.44 ± 5.82^abc^ | 6.31 ± 0.41 | 41.17 ± 1.22^a^ | 5.27 ± 0.42^b^ | 27.64 ± 1.80 | 22.26 ± 5.39^ab^ |
| ***C. claroideum*** | 0.25 ± 0.01 | 119.65 ± 2.92^a^ | 6.77 ± 0.23 | **38.32 ± 2.07^b^** | **11.58 ± 1.21^a^** | 29.36 ± 2.08 | 28.81 ± 3.98^ab^ |
| ***C. etunicatum*** | 0.25 ± 0.00 | 112.88 ± 5.41^abc^ | 7.06 ± 0.36 | **36.68 ± 0.91^b^** | 7.86 ± 1.78^ab^ | 34.96 ± 1.42 | 33.18 ± 7.66^a^ |
| ***F. mosseae*** | 0.25 ± 0.01 | 103.67 ±6.44^abc^ | 6.62 ± 0.34 | **34.08 ± 0.90^b^** | 8.34 ± 1.92^ab^ | 32.55 ± 1.95 | 13.54 ± 4.46^ab^ |
| ***G. rosea*** | 0.26 ± 0.01 | 91.31 ± 3.70^c^ | 6.30 ± 0.26 | **35.46 ± 0.83^b^** | 5.60 ± 0.60^b^ | 29.45 ± 1.49 | 12.96 ± 0.84^ab^ |
| ***S. calospora*** | 0.27 ± 0.01 | 116.35 ± 7.33^ab^ | 7.51 ± 0.32 | 39.15 ± 0.74^ab^ | 8.85 ± 1.82^ab^ | 33.90 ± 2.86 | 30.66 ± 4.66^ab^ |
| ***S. constrictum*** | 0.26 ± 0.01 | 94.42 ± 3.70^bc^ | 6.97 ± 0.35 | 37.47 ± 1.15^ab^ | 4.05 ± 0.44^b^ | 34.35 ± 2.57 | 10.79 ± 2.53^b^ |
| ***R. intraradices*** | 0.27 ± 0.01 | 97.78 ± 1.80^abc^ | 6.77 ± 0.40 | **35.30 ± 0.86^b^** | 3.73 ± 0.62^b^ | 31.09 ± 2.06 | 13.78 ± 2.85^ab^ |
| ***R. irregularis*** | 0.26 ± 0.01 | 94.18 ± 4.52^bc^ | 6.78 ± 0.27 | **34.98 ± 0.99^b^** | 6.84 ± 0.77^ab^ | 30.88 ± 1.93 | 26.77 ± 4.72^ab^ |
| **Natural community** | 0.25 ± 0.01 | 98.77 ± 2.79^abc^ | 6.76 ± 0.28 | 38.04 ± 0.86^ab^ | 4.96 ± 0.65^b^ | 29.61 ± 2.34 | 10.89 ± 1.97^b^ |
